# Impella insertion for residual aortic dissection

**DOI:** 10.1002/ccr3.4674

**Published:** 2021-08-21

**Authors:** Hisashi Yoshida, Yuki Ichihara, Ryogo Hoki, Hiroshi Niinami

**Affiliations:** ^1^ Department of Cardiovascular Surgery Tokyo Women’s Medical University Tokyo Japan

**Keywords:** aortic dissection, Impella, post‐cardiotomy cardiogenic shock

## Abstract

Acute aortic dissection with coronary malperfusion is a life‐threatening disease, resulting in demanding postoperative management. We report a successful insertion of percutaneous heart pump Impella through the intact true lumen in a patient with residual aortic dissection after the graft replacement. Careful evaluation of the access site and the Impella size selection is required.

## Graphical abstract

Percutaneous heart pump Impella could be inserted through the true lumen in patients with type A aortic dissection after the ascending and total aortic arch graft replacement.

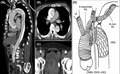

## 1 INTRODUCTION

Low cardiac output syndrome due to cardiogenic shock remains a leading cause of death following open‐heart surgery. ^1^Acute aortic dissection with coronary malperfusion is a life‐threatening disease, resulting in demanding postoperative management. The Impella (Abiomed, Danvers, MA, USA) is a percutaneous ventricular assist device associated with favorable survival outcomes in patients with post‐cardiotomy cardiogenic shock (PCCS),^2^, ^3^, ^4^ though little is known about indications for its use in patients with aortic dissection. We here report a successful insertion of percutaneous heart pump Impella through the intact true lumen in a patient with residual aortic dissection after the graft replacement and its recovery from the post‐cardiotomy cardiogenic shock.

## 2 PRESENTATION OF CASE

**FIGURE 1:**
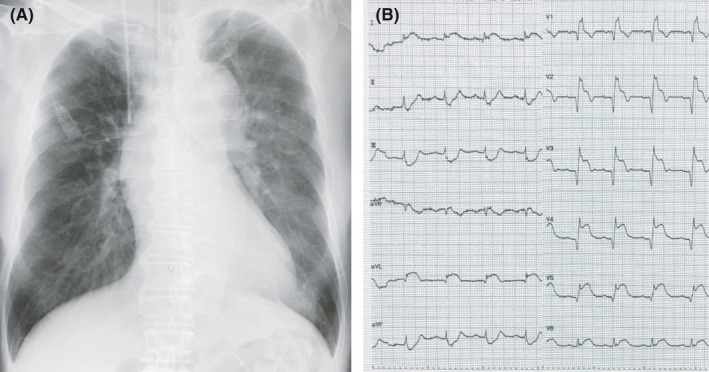
(A) Preoperative chest X‐ray showing a widened superior mediastinum and slight lung congestion. (B) Preoperative ECG showing ST‐segment elevation from the V1 to V6 leads

**FIGURE 2:**
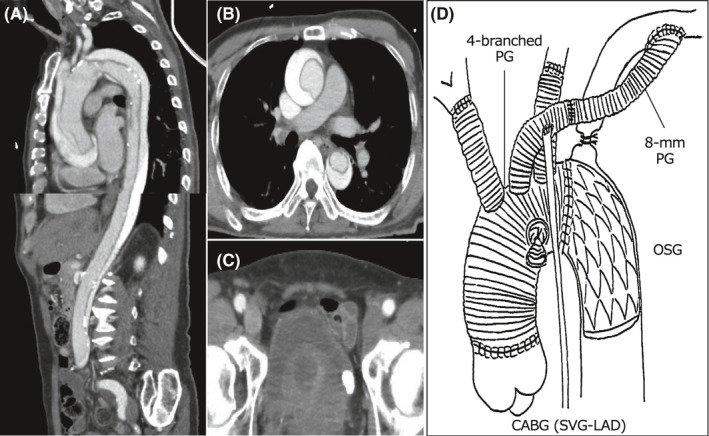
(A‐C) Preoperative computed tomography (CT) imaging. (A) Sagittal and (B) axial images showing type A acute aortic dissection extending to terminal aorta with patency of false lumen. (C) Axial images showing both femoral arteries appeared to be intact. (D) Postoperative schema of total aortic arch replacement with four‐branched prosthetic graft (PG) and open stent graft (OSG) insertion, and coronary artery bypass grafting (CABG) with saphenous vein graft (SVG) to the left anterior descending coronary artery (LAD). An 8‐mm PG was interposed between the branch of the 8‐mm aortic graft and left axillary artery. The native left subclavian artery was ligated

**FIGURE 3:**
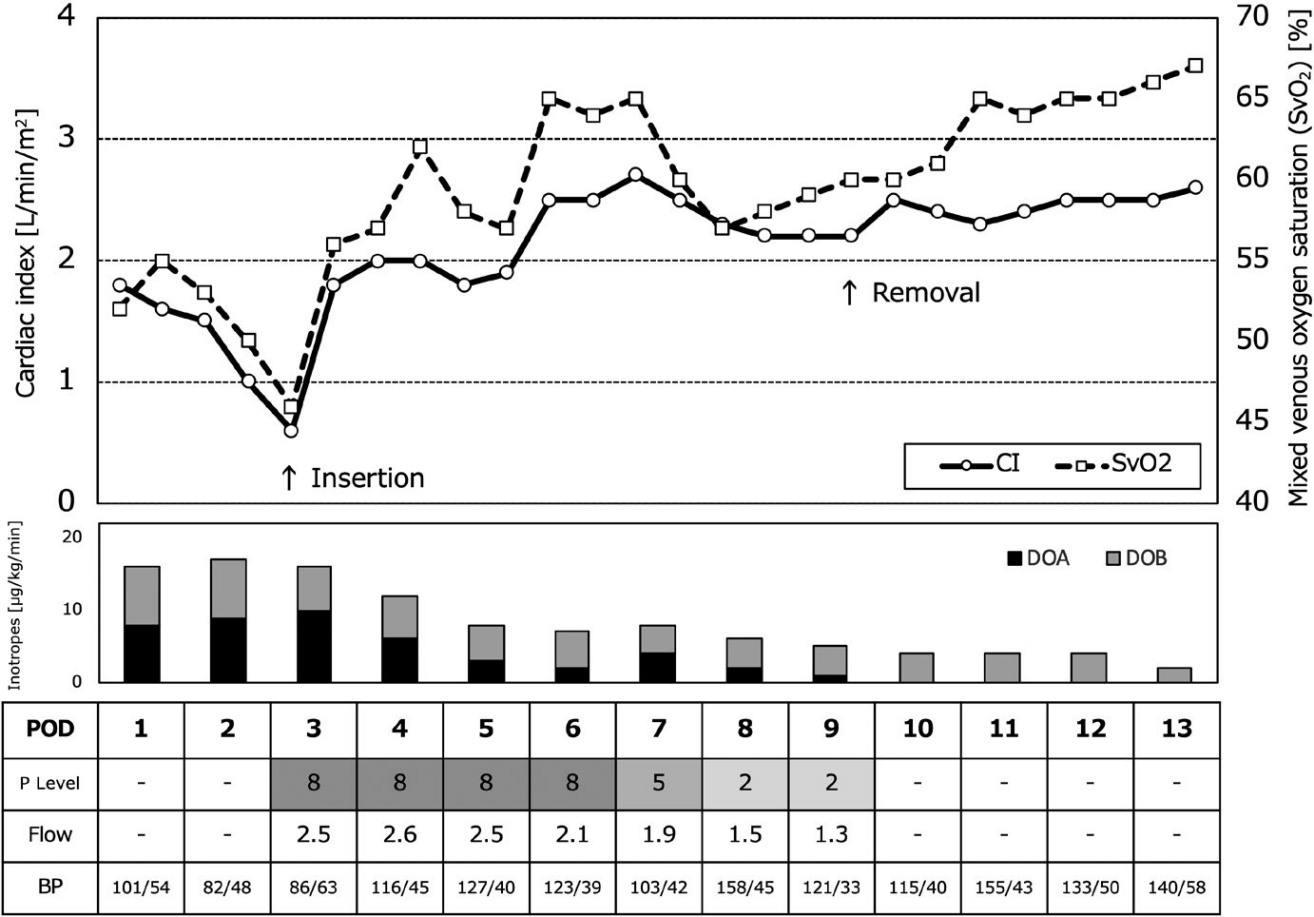
Postoperative clinical course before and after insertion of Impella 2.5. The cardiac index (CI) value and the mixed venous oxygen saturation (SvO_2_) in the Swan‐Ganz catheter were improved. DOA, dopamine; DOB, dobutamine; POD, postoperative day; P level, support level of Impella; Flow, estimated blood flow with Impella, BP, blood pressure

A 72‐year‐old male developed sudden chest pain and was urgently brought to our institute in shock status. Chest X‐ray showed a widened superior mediastinum with slight lung congestion (Figure 1A) and electrocardiogram findings revealed ST‐segment elevation from the V1 to V6 leads (Figure 1B). Transthoracic echocardiography showed an intimal flap in the ascending aorta with moderate aortic regurgitation; furthermore, a globally reduced left ventricular ejection fraction (LVEF) of 35% was noted. Computed tomography (CT) revealed Stanford type A acute aortic dissection involving the brachiocephalic artery including the right axillary artery, extending to the terminal aorta (Figure 2A‐C). Our heart team decided to perform an emergency operation. Cardiopulmonary bypass perfusion was made via a prosthetic graft (Triplex 8 mm; Vascutek Terumo, Tokyo, Japan) anastomosed to the left axillary artery because the dissection of the right femoral artery was found in contrast to the preoperative CT findings. Ascending and total arch graft replacement (Hemashield Platinum Woven Double Velour, angled 4 brarch graft, 28 mm; Getinge) with open stent graft (J Graft FROZENIX, 29 mm/60 mm; Japan Lifeline) insertion were performed. Non‐coronary cusp was prolapsed due to the dissection; however, the approximation technique with BioGlue (CryoLife Inc) led to the prevention of aortic regurgitation. Intraoperative transesophageal echocardiography showed severely reduced anteroseptal left ventricular systolic function; thus, coronary artery bypass grafting with a saphenous vein graft to the left anterior descending artery was added (Figure 2D). The cardiopulmonary bypass was weaned with high‐dose inotropes support. Postoperative clinical findings are shown in Figure 3. On postoperative day (POD) 2, cardiac index gradually decreased to 1.0 L/min/m^2^ despite further increasing inotropic support and the PCCS was developed. Coronary angiography showed the bypass graft was patent. We decided the induction with an Impella 2.5, which was inserted via the non‐affected left femoral artery in a traditional manner under fluoroscopy guidance. No adverse event associated with the insertion of the device as well as the guiding wire was found. Over the following 6 days, significant clinical improvement was demonstrated and the Impella was removed on POD9 without any complications. Hemodynamics remained continuously stable free from inotropes, and native heart recovery with an LVEF of 47% was identified by TTE at 30 days after removal. Unfortunately, the patient died from acute respiratory distress syndrome due to the sudden hemopneumothorax on POD88.

## 3 DISCUSSION

Use of an Impella provides hemodynamic stability and can rapidly reverse the death spiral of cardiogenic shock in a less‐invasive manner,^3^ though abundant caution is needed for cases with vascular disorders such as aortic aneurysm or dissection.^5^ In the present case, extracorporeal membrane oxygenation and intra‐aortic balloon pumping were clearly contraindicated. We carefully examined the access site and inserted an Impella 2.5 via the true lumen of the left femoral artery. Were that artery not available, we had attempted to insert via the left axillary artery through the replaced prosthetic graft. It might have been chosen as a first choice in terms of the rapid and easy access; however, it has recently been reported that the longer implant duration of the Impella through the axillary artery correlates strongly with thrombus formation.^6^ Therefore, we thought that the insertion from this area in the present case is not always reasonable in consideration of the impact of the prosthetic graft accompanying with the anastomosis of the bypass graft in a middle way.

Regarding size selection, the Impella CP or 5.0 could have been the best size in terms of the adequate systemic flow for the patient's body surface area (1.7 m^2^). On the other hand, the Impella 2.5 has the smallest pump size with 12 Fr, thus, it was considered capable of passing across the dissecting lumen. The improvement of the hemodynamics after insertion of the Impella 2.5 demonstrated that our strategy would have been suitable including the size selection and access site.

## 4 CONCLUSION

To the best of our knowledge, this is the first report of Impella use for a patient with residual aortic dissection and it suggests that the device could be inserted through the true lumen in case. Nevertheless, careful evaluation of the access site and Impella size selection are required.

## CONFLICTS OF INTEREST

The authors have no conflicts of interest to declare.

## AUTHOR CONTRIBUTIONS

All authors participated in clinical care of the patient. HY prepared and edited the figures. RH, YI, and HN were involved in management of the patient. YI coordinated writing of the manuscript.

## ETHICAL APPROVAL

Written informed consent was obtained from the patient's family for publication of this case report.

## Data Availability

The authors confirm that the data supporting the findings of this study are available within the article.
